# Characteristics of CD4+CD25+ regulatory T cells in the peripheral circulation of patients with head and neck cancer

**DOI:** 10.1038/sj.bjc.6602407

**Published:** 2005-02-15

**Authors:** C Schaefer, G G Kim, A Albers, K Hoermann, E N Myers, T L Whiteside

**Affiliations:** 1 University of Pittsburgh Cancer Institute, University of Pittsburgh School of Medicine, Pittsburgh, PA 15213, USA; 2 Department of Head and Neck Surgery, University Hospital of Mannheim, Mannheim, Germany; 3 Department of Otolaryngology, University of Pittsburgh School of Medicine, Pittsburgh, PA 15213, USA; 4 Department of Pathology, University of Pittsburgh School of Medicine, Pittsburgh, PA 15213, USA; 5 Department of Immunology, University of Pittsburgh School of Medicine, Pittsburgh, PA 15213, USA

**Keywords:** head and neck cancer, CD4+CD25+ T cells, regulatory T cells (T_reg_), CD4+ T-cell subsets

## Abstract

Patients with squamous cell carcinoma of the head and neck (SCCHN) have depressed antitumour immunity. The presence of CD4+CD25+ (T_reg_) cells in these patients might be, in part, responsible for downregulation of antitumour immune responses. To evaluate the frequency and characteristics of T_reg_ in the peripheral circulation of patients with SCCHN, we used multicolour flow cytometry. Expression of CCR7, CD62L, *ζ* chain and Annexin V binding to T_reg_ and non-T_reg_ CD4+ lymphocyte populations were evaluated. T_reg_ were confirmed to be Foxp3+ and GITR+. The T_reg_ frequency was significantly elevated in patients with active disease and those with no evidence of disease (NED) following curative therapies. Both T_reg_ and non-T_reg_ CD4+ T cells in patients were significantly enriched in CCR7^−^ and CD62L^−^ cell subsets. Although T_reg_ in patients contained a higher proportion of double negative (CCR7^−^CD62L^−^) cells, the majority of T_regs_ were CCR7^−^CD62L+. The proportion of Annexin V+CD4+ T cells was higher in patients (*P*<0.00005) than normal controls (NC), and T_reg_ were significantly more sensitive to apoptosis than non-T_reg_ in patients and NC. Expression of *ζ* was reduced in all subsets of CD4+ T cells obtained from patients *vs* NC. The data suggest that T_reg_ in patients with SCCHN largely contain T cells with the ‘effector’ phenotype, which bind Annexin V and have low *ζ* expression, consistent with their activation state and a rapid turnover in the peripheral circulation.

Immune tolerance to self-antigens is essential for the prevention of autoreactivity and autoimmune diseases. Currently, a subset of CD4+ T cells expressing CD25, the IL-2 receptor *α*-chain, and referred to as regulatory T cells (T_reg_), is considered to be responsible for the control of autoreactive lymphocytes (Shevach, 2000). Substantial experimental evidence supports the regulatory role of CD4+CD25+ T cells in animals and man. Thus, in rodents, depletion of T_reg_ resulted in the development of autoimmune diseases and enhanced immune responses against alloantigens and tumour-antigens, leading to rejection of transplanted tumours by the host immune system (Shimizu *et al*, 1999; Sakaguchi *et al*, 2001; Sutmuller *et al*, 2001). This finding was not unexpected, as a majority of known tumour-associated antigens (TAA) are altered or overexpressed self-antigens. Therefore, CD4+CD25+ T_reg_ are a likely candidate for downregulation of immune responses to these TAA. In man, T_reg_ have been described to be present in increased proportions among tumour-infiltrating lymphocytes (TIL) and in the peripheral circulation of patients with various malignancies (Woo *et al*, 2001; Liyanage *et al*, 2002; Woo *et al*, 2002; Wolf *et al*, 2003). Based on phenotypic characteristics, this population of lymphocytes accounts for 5–10% of all CD4+ T cells in the peripheral circulation of healthy individuals (e.g., Wolf *et al*, 2003), but in patients with cancer, peripheral mononuclear cells (PBMC) may contain up to 25–30% of T_reg_ (Woo *et al*, 2002).

Patients with squamous cell carcinoma of the head and neck (SCCHN) have depressed antitumour immune responses, and tumour progression or recurrence is associated with particularly severe immune dysfunction (Whiteside, 2004). While several distinct mechanisms have been suggested to explain immune unresponsiveness in these patients, including the presence of tumour-derived inhibitory factors (e.g., PGE_2_), T-cell apoptosis, inhibitory cytokines (e.g., IL-10) or excess of suppressive macrophages, the presence of T_reg_ at a high frequency could also contribute to lymphocyte dysfunction, which is characteristically and consistently observed in patients with SCCHN (Reichert *et al*, 1998; Hoffmann *et al*, 2002; Reichert *et al*, 2002).

The CD4+ T-cell subset of lymphocytes was previously studied by us in patients with SCCHN (Kuss *et al*, 2003; Kuss *et al*, 2004a). We found that the absolute count of circulating CD4+ T cells was significantly decreased in these patients (Kuss *et al*, 2004a). While the pool of naïve CD4+CD45RO-CD27+ T cells was significantly smaller in patients than in age-matched normal controls (NC), the pool of memory CD4+CD45RO+ T cells was significantly expanded (Kuss *et al*, 2003). Thus, based on the lymphocyte count, their phenotype and the T-cell receptor excision circle (TREC) analysis (Kuss *et al*, 2004b), it appears that the significant paucity of naïve and excess of memory CD4+ T cells characterises the subset of CD4+ T cells in SCCHN patients. However, phenotypic characteristics or distribution of CD4+CD25 T_reg_ among these CD4+ cell subsets is not known.

In the present study, we investigated whether the circulating pool of CD4+CD25+ T_reg_ in patients with SCCHN was expanded relative to that in NC and evaluated phenotypic and functional characterisitics of T_reg_. The hypothesis tested predicted that PBMC of these patients might be enriched in CD4+CD25+ T_reg_, which, by virtue of downregulating antitumour immune functions, could contribute to the progression or recurrence of head and neck cancer.

## MATERIALS AND METHODS

### Patients and controls

Patients with head and neck cancer (HNC) were seen at the Outpatient Otolaryngology Clinic, University of Pittsburgh Medical Center (UPMC) between February and December 2003. Blood samples were obtained from consecutive patients who signed an informed consent form approved by the Institutional Review Board. Normal healthy donors (NC), aged from 45 to 70 years, were recruited among the laboratory personnel and asked to sign the consent form prior to donating blood. The study included 24 patients with HNC and 17 NC. The clinicopathologic characteristics of the patients included in the study are shown in Table 1. The cohort included 16 male and eight female subjects aged from 46 to 84 years. All patients had histologically proven SCCHN, with six cancers originating in the larynx, nine in the oral cavity, two in the oropharynx and seven in the hypopharynx. Nine out of 24 patients had T3 or T4 disease, and 13 out of 24 had nodal metastases. At the time of the blood draw, 14 patients showed no evidence of disease (NED), having previously received curative therapies, and 10 patients had active disease (AD; presurgery).

### Cell isolation

Venous blood obtained from patients or controls (20–30 ml) was delivered to the laboratory within 2 h of phlebotomy. Peripheral mononuclear cells were isolated from venous blood by density gradient sedimentation on Ficoll-Hypaque. Cells recovered from the gradient interface were washed twice in medium, counted and immediately used for staining.

### Antibodies

The following monoclonal antibodies (mAbs): anti-CD3-ECD (UCHT1), anti-CD4-PE (13B8.2), anti-CD8-PC5 (SFCI21Thy2D3), anti-CD25-PC5 (B1.49.9), anti-CD62L-ECD (DREG56) and the respective isotypes used as negative controls for surface staining were all purchased from Beckmann Coulter (Miami, FL, USA). Anti-CCR7-FITC mAb was purchased from R&D Systems Inc., Minneapolis, MN USA. For intracellular detection of TCR *ζ* chain, anti-CD247-FITC (6B10.2) obtained from Santa Cruz Biotechnology, Inc., CA, USA was used. Unlabelled polyclonal anti-Foxp3 Abs and the secondary labelled Abs were purchased from Abcam, Ltd., Cambridge, MA, USA. Carboxyfluorescein-conjugated mAb to glucocorticoid-induced TNF receptor (GITR) was purchased from R & D Systems.

### Annexin V apoptosis detection kit

The Annexin V-FITC Apoptosis Detection Kit was purchased from BD PharMingen and used as recommended by the manufacturer.

### Cell staining

Cells were resuspended in phosphate-buffered saline (PBS), containing 0.1% BSA and 0.1% NaN_3_ to the final concentration of 2 × 10^6^ ml^−1^. Cells were stained for flow cytometry as previously described (Kuss *et al*, 2003). Briefly, optimal working dilutions of all Abs used for staining were first determined by titrations with normal PBMC. Checkerboard titrations were used to determine optimal dilutions of primary and secondary Abs used for indirect staining (Foxp3). For surface staining, PBMC were incubated with the optimal dilution of each mAb for 25 min at 4°C in the dark, washed twice with PBS containing 0.1% (w v^−1^) bovine serum albumin (BSA) and 0.1% (w v^−1^) N_a_N_3_ and finally fixed by adding 2% (v v^−1^) paraformaldehyde (PFA) in PBS. For intracellular staining (TCR *ζ* chain and Foxp3), surface staining for T-cell markers was followed by two washes with the PBS/BSA/NaN_3_ buffer and by subsequent fixation of the PBMC in 2.5% PFA for 10 min at room temperature in the dark. After another wash with the same buffer, PBMC were permeabilised with saponin (0.1% v v^−1^ in BSA) and washed with cold saponin solution. Next, anti-*ζ* or anti-Foxp3 mAb or IgG1-FITC isotype was added to the cells. After incubation for 25 min at 4°C in the dark, the cell suspension was washed again with 0.1% saponin, followed by another wash with the PBS/BSA/NaN_3_ buffer or by a secondary Ab in the case of Foxp3. The cells were finally fixed with 2% PFA in PBS. Stained samples were immediately analysed by flow cytometry.

### Flow cytometry analysis

Flow cytometry was performed on a Coulter Epics XL Flow-Cytometer. Gating strategy used to identify the Anx+PI- populations of lymphocytes was previously described (Bauernhofer *et al*, 2003). To identify T_reg_ populations, the gate was set to acquire CD4+CD25^high^ T cells. The subsequent data analysis was performed using the Coulter EXPO 32vl.2 analysis software.

### Statistical analysis

Statistical analyses were performed using the Student's *t*-test, and *P*-values of <0.05 were considered significant.

## RESULTS

### Proportions of T_reg_ in the circulation of patients with SCCHN and controls

**Figure 1:**
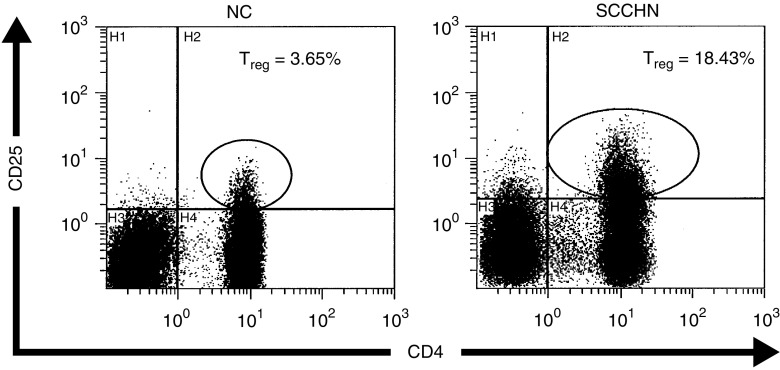
Enrichment in CD4+CD25+ T_reg_ in PBMC of a patient with SCCHN relative to a normal control. Flow cytometry was performed as described in Materials and Methods. The gate was set to acquire bright CD4+CD25+ T cells. Isotype controls were used to set the cut-off for CD25+ *vs* CD25^−^ cells.

**Figure 2:**
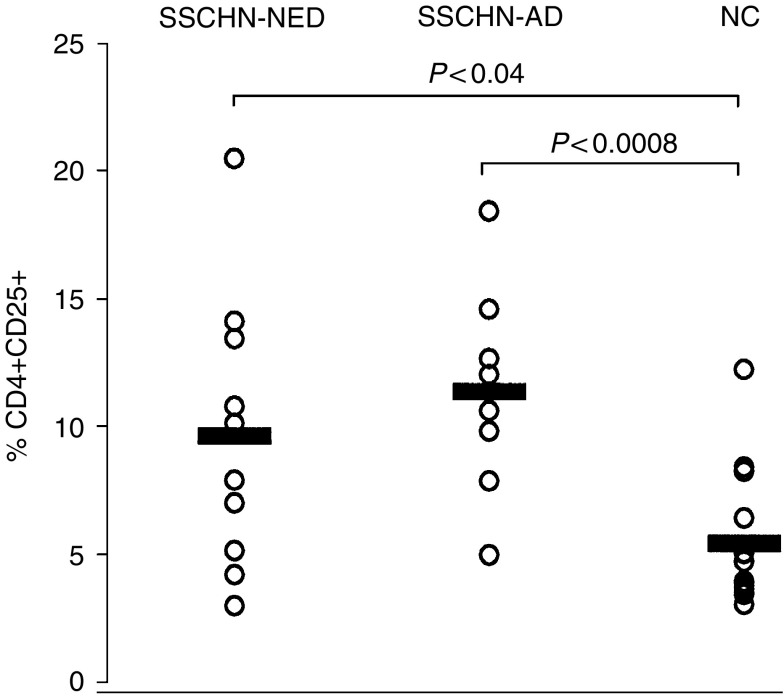
Frequencies of CD4+CD25+ T_reg_ in the circulation of patients with SCCHN and NC. The patients with AD at the time of phlebotomy were not different from those with NED (*P*>0.2). However, the NED and AD patient cohorts were significantly different from NC (*P*<0.04 and *P*<0.0008, respectively).

Flow cytometry analysis indicated that the patients had significantly higher percentages of CD4+CD25+ T_reg_ in the circulation than NC (10.1±4.7 *vs* 5.4±2.7%). Representative flow cytometry results for one patient and one normal control are shown in Figure 1. This finding is consistent with the data reported for patients with other epithelial cancers (Woo *et al*, 2001; Liyanage *et al*, 2002). Among the cohort of 19 patients, eight had AD at the time of blood draws for this study, and 11 had NED following curative therapies (Table 1). No significant difference in the proportion of T_reg_ was observed between these two patient cohorts, as illustrated in Figure 2. However, the percentage of T_reg_ was significantly higher in patients with AD (*P*<0.0008) or NED (*P*<0.04) than that in NC.

Although we gated on CD4+CD25+^high^ T cells, which is considered to be the phenotype of T_reg_, it was necessary to confirm their identity through expression of Foxp3 or GITR markers (Ramsdell, 2003; Ronchetti *et al*, 2004). To this end, we developed a flow-cytometry-based method with commercially available Abs to measure expression of these markers on CD4+CD25^high^ T cells. In five patients with SCCHN, PBMC were found to contain an average of 7±1.5% of CD4+CD25+ T cells, 5±1.0% of CD4+CD25+ GITR+ T cells and 6.0±1.3% of CD4+CD25+Foxp3+ T cells (Table 2). These data confirm that the large majority of CD4+CD25^high^ T cells express Foxp3 or GITR, the markers associated with T_regs_.

### Expression of CCR7 on CD4+ T-cell subsets

**Figure 3:**
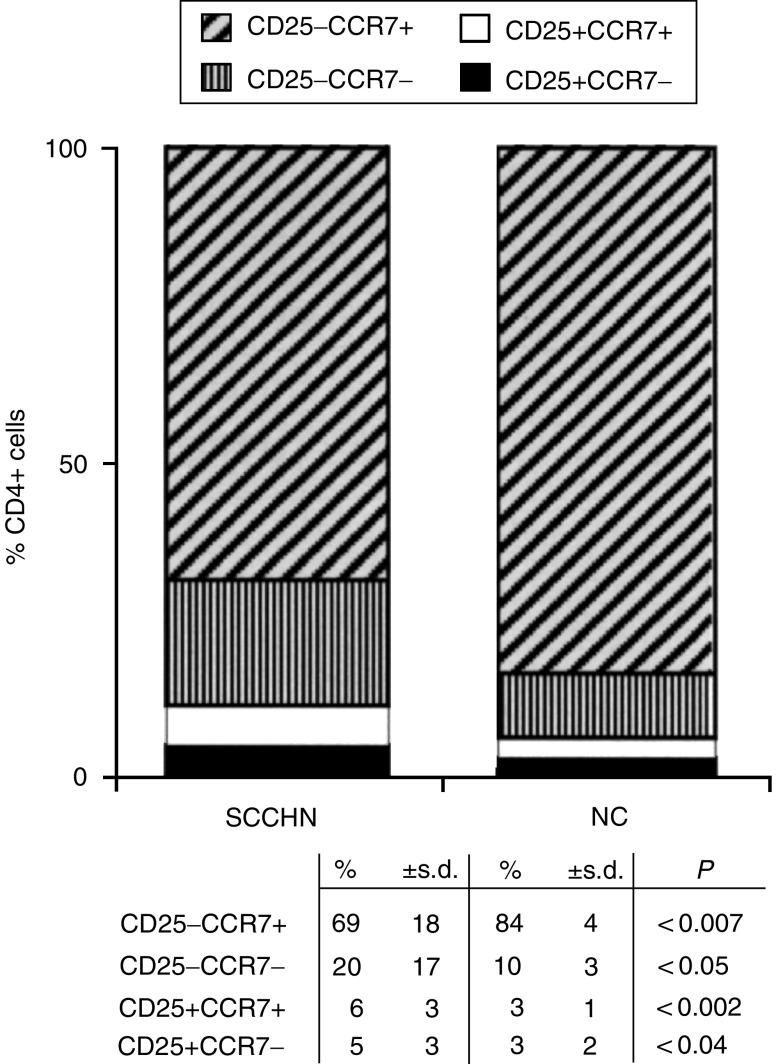
Altered distribution within the CD4+ T-cell subset of cells expressing CD25 and CCR7 markers in patients with SCCHN relative to NC. Note a significant decrease in CD4+CD25^−^CCR7+ and an enrichment in CD4+CD25^−^CCR7^−^, CD4+CD25+CCR7+ and CD4+CD25+CCR7^−^ T cells in the circulation of patients with SCCHN relative to NC.

Expression of the chemokine receptor CCR7 on the surface of lymphocytes is associated with the acquisition of a migratory phenotype (Sallusto *et al*, 1999; Campbell *et al*, 2001). Thus, mature CD4+ T cells expressing CCR7 are able to migrate to secondary lymphoid organs, while those lacking CCR7 are effector cells. We, therefore, studied expression of this receptor on CD4+ T cells of patients and NC, with special attention focused on the subset of T_reg_. The expectation was that the percent of CD4+CD25+CCR7+ cells would be lower in patients than NC, based on our report documenting an enrichment in T_reg_ at tumour sites and tumour-involved lymph nodes relative to the peripheral circulation (Albers *et al*, 2004). Instead, as shown in Figure 3, the percentages of both CCR7+ and CCR7^−^ T_reg_ were significantly increased (*P*<0.002 and *P*<0.04) in the circulation of patients *vs* NC. Conversely, among non-T_reg_ CD25^−^CD4+ cells, CCR7+ cells were significantly lower in patients than NC (*P*<0.007), and significant expansion of the CCR7^−^ subset was evident (Figure 3). These expanded CCR7^−^ cells are presumably terminally differentiated effector T cells (Sallusto *et al*, 1999). The significant shifts observed in the content of CCR7^−^ and CCR7+ cells among the CD4+ subsets of lymphocytes in patients with SCCHN are suggestive of their distinct distribution between blood *vs* tissues and perhaps of differences in the activation state or turnover rate among functionally distinct subsets of lymphocytes.

### Expression of CD62L on CD4+ T-cell subsets

**Figure 4:**
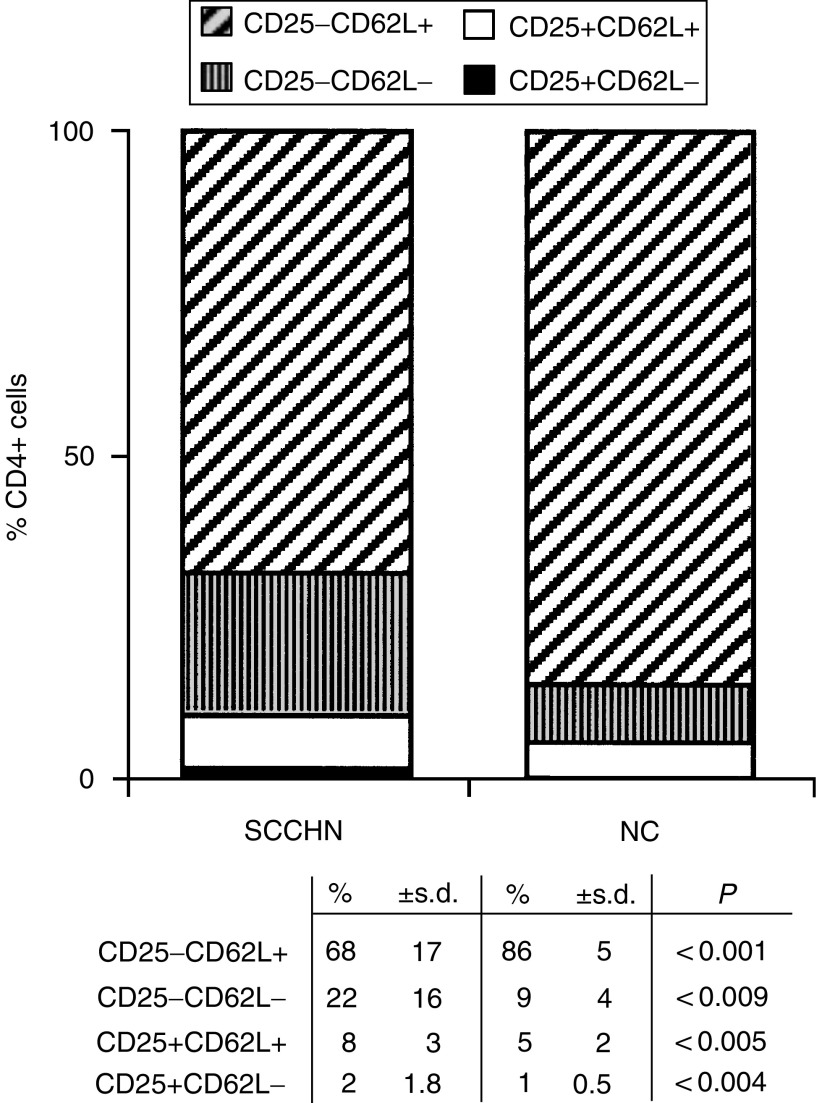
Changes in the distribution within the CD4+ T-cell subset of cells expressing CD25 and CD62L markers. Note a significant decrease in CD4+CD25^−^CD62L+ and an enrichment in CD4+CD25^−^CD62L^−^, CD4+CD25+CD62L+ and CD4+CD25+CD62L^−^ T cells in the circulation of patients relative to NC.

Like CD27 antigen, L-selectin (CD62L) expression is lost on mature, more differentiated T cells, which are responsible for helper/suppressor functions performed by CD4+ T cells. For this reason, we expected to find an increased percentage of CD4+ T cells with the CD62L^−^ phenotype in the peripheral circulation of patients with SCCHN. As seen in Figure 4, this indeed was the case, and the expansion of CD62L^−^ non-T_reg_ CD4+ T in patients with SCCHN was accompanied by a significant concomitant decrease in the percent of CD4+CD25^−^CD62L+ cells. In contrast, among CD25+CD4+ T_reg_, enrichment in CD62L+ cells (*P*<0.005 relative to NC) exceeded that of CD62L^−^ cells. These observations imply that T_reg_ in the circulation of patients might be at a different maturation or activation stage than their normal counterparts. Further, the proportion of CD4+CD25^−^ T cells that are CD62L+, that is, belong to the naive pool, is decreased in the patients with a concomitant enrichment in effector CD4+ T cells.

### Concomitant expression of CCR7 and CD62L on T_reg_

**Figure 5:**
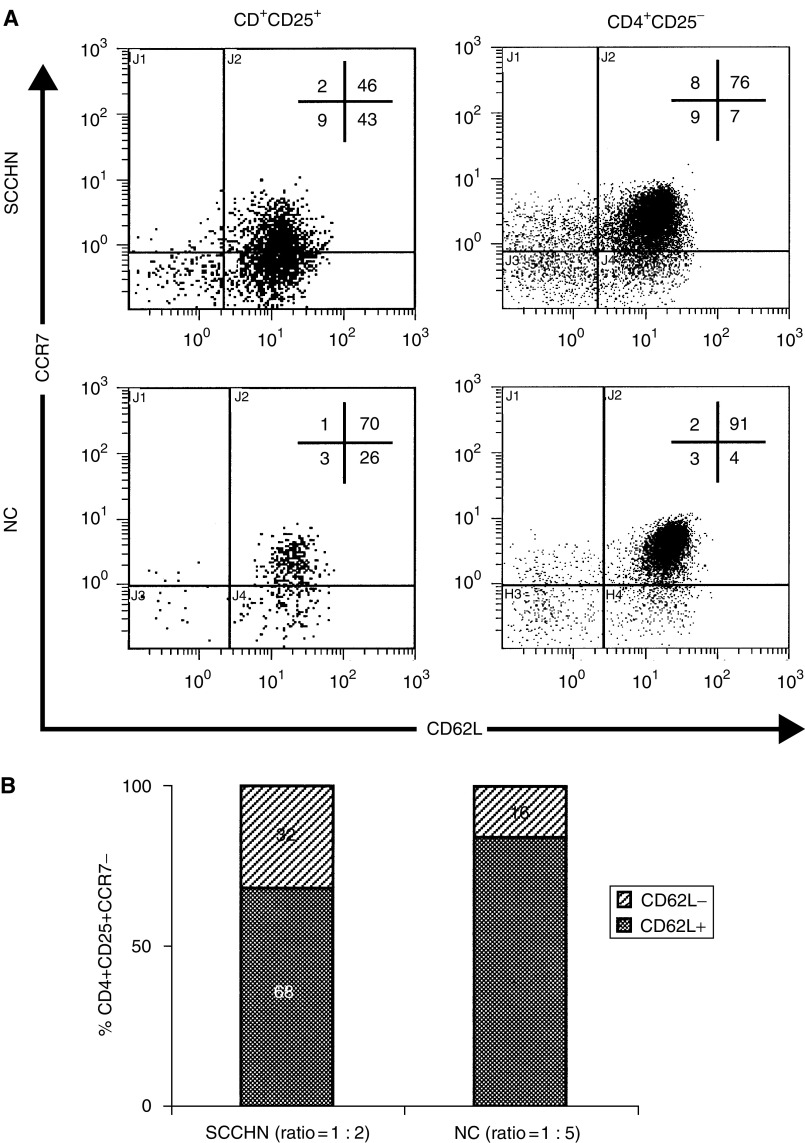
In (**A**), multicolour flow cytometry of PBMC obtained from a representative patient with SCCHN shows an increased proportion of CCR7^−^CD62L+ T_reg_ relative to non-T_reg_ CD4+CD25^−^ cells and to NC. In (**B**), expansion of CD62L^−^ T cells within the subset of CCR7-CD25+CD4+ T_reg_ in patients with SCCHN relative to NC. The gate was set on CD4+ T cells and subsets of CD25+CCR7^−^CD62L^−^ *vs* CD25+CCR7^−^CD62L+ were quantitated by four-colour flow cytometry.

T_reg_ in patients with SCCHN contained a higher proportion of CCR7^−^ and CD62L^−^ cells than NC (Figures 3 and 4). A loss of CCR7 and/or CD62L markers accompanies maturation, activation or terminal differentiation of T cells (Sallusto *et al*, 1999; Campbell *et al*, 2001). We were, therefore, especially interested in a concomitant loss of expression of both these markers on T_reg_ in the peripheral circulation of patients *vs* controls. Figure 5A is an example of the four-colour flow cytometry analysis performed with cells of a representative patient and a normal control. It shows that the percentage of T_reg_ is higher in the patient than in NC and that substantially more T_reg_ are CCR7^−^CD62L+ in the patient (43%) *vs* NC (26%). Concomitantly, the patient has fewer T_reg_ that are CCR7+CD62L+ than NC. Thus, T cells with the CCR7^−^CD62L+ phenotype are the major T_reg_ subset in patients with SCCHN. To further focus on this subset, we next examined CD62L expression on CD4+CD25+CCR7^−^ cells in patients and NC. As shown in Figure 5B, a significant difference (*P*<0.03) was observed in the ratio of double negative (CCR7^−^CD62L^−^)/(CCR7^−^CD62L+) T_reg_ between the patients (1 : 2) and NC (1 : 5).

In aggregate, these data are consistent with the conclusion that among T_reg_ those with CCR7^−^CD62L+ and CCR7^−^CD62L^−^ phenotypes are enriched in the circulation of patients with SCCHN relative to NC. This finding implies that more T_reg_ in the circulation of patients with SCCHN than in NC are terminally differentiated and are undergoing rapid maturation and turnover.

### Annexin V binding to the subsets of CD4+ T cells

**Figure 6:**
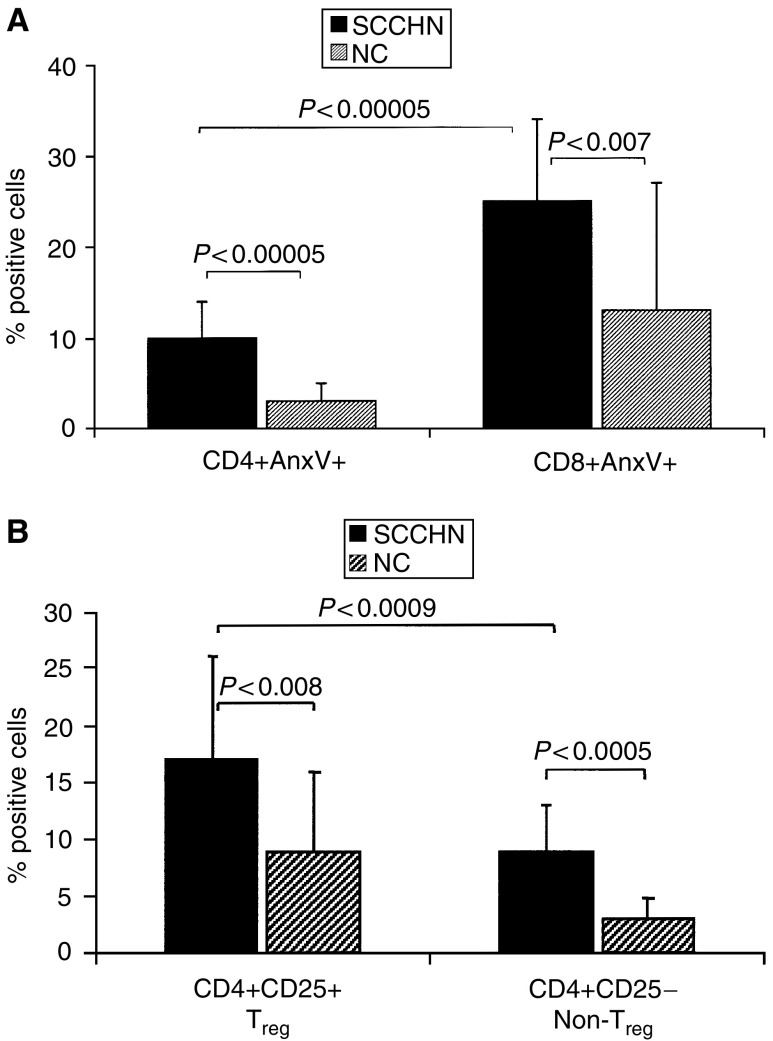
Annexin V (Anx V) binding to T cells. In (**A**), binding of Anx V to CD4+ and CD8+ T-cell subsets in patients with SCCHN and in NC. In (**B**), Anx V binding to T_reg_ (CD4+CD25+) *vs* non-T_reg_ (CD4+CD25^−^) T cells in patients with SCCHN and NC.

We have previously reported a decrease in absolute CD4+ T-cell counts in patients with SCCHN relative to age-matched NC (Kuss *et al*, 2004a). In the cohort of patients studied here, the mean absolute CD4+ T-cell count was 610±350 mm^−3^, which is significantly lower than the normal mean count of 1005±360 mm^3^ (Kuss *et al*, 2004a). It was, therefore, important to determine whether the low absolute counts of circulating CD4+ T cells could be explained by their apoptosis. To this end, Annexin V (Anx V) binding to CD8+ and CD4+ circulating T cells was evaluated by flow cytometry. In agreement with previously published results (Hoffmann *et al*, 2002), we observed significantly lower percentage of Anx V+CD4+ than CD8+ T cells in SCCHN patients and in NC (Figure 6A). Although Annexin binding to CD4+ T cells was significantly higher (*P*<0.00005) in patients than in NC (Figure 6A), the overall proportion of CD4+ T cells undergoing apoptosis was low, not exceeding 10% in the patients’ circulation.

We next examined Anx V binding to the CD4+ T-cell subsets, including CD4+CD25+ and CD4+CD25^−^ cells, to determine whether T_reg_ are more or less sensitive to apoptosis than non-T_reg_. As shown in Figure 6B, Anx V bound to 17.4% of T_reg_ and 9% of non-T_reg_ in patients with SCCHN. In NC, 8.6% of T_reg_ *vs* 3% of non-T_reg_ bound Anx V. Anx V binding was significantly higher among T_reg_ than non-T_reg_ in the circulation of patients as well as NC (*P*<0.0009). This observation suggests that T_reg_ are more rapidly cleared from the peripheral circulation than other CD4+ cells, perhaps as a result of their activation and migration to tissues to be utilised in regulatory activities. This rapid turnover of T_reg_ relative to non-T_reg_ among CD4+ T cells might be a manifestation of activation induced cell death (AICD).

### Expression of *ζ* in T_reg_ of patients with SCCHN

Expression of *ζ* chain in CD3+ T cells or their subsets is considered to be a measure of their functional status, as it determines the ability of T cells to signal upon TCR engagement (Whiteside, 2004). Using flow cytometry, we initially determined that CD8+ T cells in our cohort of patients with SCCHN had lower expression of *ζ* (log MFI=2.8±0.6) than CD4+ T cells (log MFI=3.4±0.7) as shown in Table 3. Although not statistically significant, this difference in the MFI was consistent with higher levels of Anx V binding to CD8+ T cells, as indicated above (Figure 6A). Among CD4+ T cells, no difference was observed in MFI for *ζ* expression between T_reg_ and non-T_reg_ (Table 3). However, consistent with a higher percentage of T_reg_ in the peripheral circulation of SCCHN patients, *ζ* expression was found to be lower in all T-cell subsets (CD4+, CD8+, non-T_reg_) in these patients as compared to NC (Table 3). Thus, we hypothesise that T_reg_, which are more numerous in patients than in NC, are responsible for downregulation of TCR-mediated signalling in CD8+ and non-T_reg_ CD4+ T cells.

## DISCUSSION

The T_reg_ subset of CD4+ lymphocytes (CD4+CD25^high^ cells) has assumed a considerable importance within the immunoregulatory network both in health and disease. These cells exert suppressive activity upon CD8+ effector and CD4+ helper T cells, and their effects appear to be dose dependent, cell-contact dependent, cytokine independent and antigen-nonspecific (Shevach, 2000; Dieckmann *et al*, 2001; Jonuleit *et al*, 2001; Ng *et al*, 2001; Taams *et al*, 2001;). However, more recent studies suggest that subpopulations of T_reg_ endowed with different regulatory functions, including suppression of TAA-specific immune responses, exist in the circulation and/or tissues of patients with cancer (Woo *et al*, 2002; Shevach, 2004). Given the emerging heterogeneity of this cell population, it is important to consider how subsets of CD4+CD25+ cells relate to other helper T cells and to disease in individuals with cancer.

The percentage and absolute numbers of CD4+CD25+ T cells were reported to be increased in the peripheral circulation of patients with various malignancies (Woo *et al*, 2001; Liyanage *et al*, 2002). In patients with SCCHN, we have recently described a significant enrichment in CD4+CD25+ T cells in the peripheral blood and particularly among tumour-infiltrating lymphocytes (Albers *et al*, 2004). However, it now appears that T_reg_ are a heterogenous population within CD4+ T cells, and that a subset of CD4+CD25+ T cells could be subdivided into different functional subsets based on expression of novel markers such as GITR, Foxp3 or neurophilin-1 (Cosmi *et al*, 2003; Ramsdell, 2003; Bruder *et al*, 2004; Ronchetti *et al*, 2004). Further, a subset of these cells could represent activated CD4 cells expressing the CD25+ receptor. Taking advantage of newly available Abs to GITR and Foxp3, we performed multiparameter flow cytometry to demonstrate that most CD4+CD25^high^ T cells in the circulation of patients with HNC express Foxp3 or glucocorticord-induced TNF receptor (GITR). While this analysis was performed in only a small subset of our patients, it offers support for the phenotypic analysis of T_reg_ subsets presented here.

The present study confirmed the enrichment in T_reg_ among PBMC of HNC patients and offered us an opportunity to determine whether patients with AD had more circulating CD4+CD25+ T cells than those with NED. Surprisingly, both cohorts had a higher frequency of T_reg_ than NC, and patients presumably cured of their disease following therapy were not different from those with tumours in respect to the proportions of T_reg_ in the periphery. This observation supports our hypothesis that lymphocyte homeostasis disrupted by the presence of tumour fails to normalise following successful therapy. Immune abnormalities, including depressed absolute lymphocyte counts (Kuss *et al*, 2004a), elevated percentages of CD8+ cells undergoing early apoptosis (Hoffmann *et al*, 2002), and signalling defects (Kuss *et al*, 2003), persist for weeks or years after curative therapies in patients with NED. It appears that the enrichment in circulating T_reg_ is also a persistent immune alteration that does not normalise following therapy. Nevertheless, it is apparent from the data (Figure 2) that as a group, SCCHN patients with AD differ most significantly from NC in the percentage of circulating CD4+CD25+ T_reg_.

The maturation status of T_reg_ and their ability to home to lymph nodes are attributes that determine their biologic activity. We have previously shown that the majority of CD4+ T cells in the circulation of patients with SCCHN have a memory phenotype (Kuss *et al*, 2003). Thus, it was not unexpected to find that CD4+ T cells in patients with SCCHN were characterised by the loss of CCR7 and L-selectin surface molecules, which are functionally associated with cell migration into tissues and serve as phenotypic markers for distinct stages of lymphocyte differentiation, respectively. The implication of this finding is that in patients, CCR7+ and L-selectin+ CD4+ T cells tend to localise to tissues. We have previously reported a significant and sometimes striking enrichment in CD4+CD25+ T cells among tumour-infiltrating lymphocytes in patients with SCCHN (Albers *et al*, 2004), and thus we hypothesise that these cells may preferentially localise to tumour sites. If so, then T_reg_ present in the circulation of SCCHN patients might be a mix of less mature (CCR7+CD62L+) lymphocytes being recruited from the naïve lymphocyte pool and of activated or terminally differentiated T_reg_, which have lost either CCR7 or CCR7 and CD62L markers. In fact, subsets of CCR7^−^ and CD62L^−^ T_reg_ are expanded in the circulation of SCCHN patients relative to NC. In addition, the ratio of more differentiated (CCR7^−^CD62L^−^) to less differentiated (CCR7^−^CD62L+) T_reg_ is altered in patients *vs* NC, because of an increase in absolute and relative numbers of more differentiated T_reg_ in patients with SCCHN. This shift in T_reg_ subsets could be a result of selective migration to tissues and accumulation at the tumour site or in tumour involved lymph nodes (Albers *et al*, 2004). Alternatively, it implies that activated T_reg_ in the patients’ circulation undergo a rapid turnover. The observed increased binding of Anx V by T_reg_ is consistent with this hypothesis and with previous reports indicating that CD4+CD25+ T cells are more susceptible to apoptosis than other CD4+ T cells (Salmon *et al*, 1994; Akbar *et al*, 1996; Taams *et al*, 2001). Such rapid turnover of T_reg_ in patients with SCCHN and other cancers (i.e., entry from the naïve pool, maturation and migration into tissues) explains their enrichment in lymphoid and tumour tissues, and the phenotype of T_reg_ remaining in the circulation. Based on the principle that lymphocyte activation is accompanied by their migration into tissues and/or AICD, reconstitution of the lymphocyte pool from naïve precursors must be quite efficient. However, even this rapid replacement does not seem to correct absolute CD4+ T lymphocyte count in patients with SCCHN, which ranged from 120 to 1227 mm^−3^, with a mean of 610±350 (s.d.) mm^−3^ in our patient cohort, and was significantly lower from the normal mean of 1005±360 mm^−3^ (*P*<0.001).

The expansion of T_reg_ in the circulation and tissues of patients with cancer suggests that these cells downregulate antitumour responses, guarding from overly vigorous responses to self-antigens. Functional evaluations of T_reg_ require their isolation and titration into functionally active tumour-specific T cells. The latter are not easily available, in humans, and are particularly difficult to generate in patients with SCCHN, where few immunogenic tumour-specific epitopes are available. For this reason, we resorted to an alternative approach dependent on the analysis of *ζ* expression, which is a signalling component of TCR in CD4+ and CD8+ T-cell subsets. The rationale for measurements of *ζ* expression was based on our previous data linking T-cell dysfunction in patients with SCCHN with the absence or low expression of the *ζ* chain (Kuss *et al*, 1999; Whiteside, 2004). In the presence of T_reg_, which presumably downregulate functions of other T cells, the level of *ζ* expression in these T cells is expected to be low, thus providing some indication about activity of T_reg_. It was interesting to observe that in patients with SCCHN, various circulating T-cell subsets had lower expression of *ζ*, as compared to their counterparts in NC. This observation is consistent with previous reports from our laboratory (Kuss *et al*, 1999). It suggests that signalling, and therefore effector or helper functions of T cells obtained from the circulation of patients with SCCHN are compromised in signalling via TCR, possibly because of the presence of T_reg_.

Finally, it has to be conceded that further analysis of the functional potential of T_reg_ will be necessary to elucidate their impact on disease progression. The current study, performed largely by using multicolour flow cytometry with a small number of blood samples obtained from patients with SCCHN is obviously limited by the availability of cells. Nevertheless, it illustrates the importance of T_reg_ in defining the immune profile of patients with cancer and emphasises the role of these cells in downregulating functions of other T-cell subsets.

## Figures and Tables

**Table 1:** Clinicopathologic features of patients with SCCHN included in the study

	***n***
**Age (years) mean (range)**	**58 (46–84)**
*Sex*	
Male	16
Female	8

Total	24

*Tumour site*	
Larynx	6
Oral cavity	9
Pharynx	2
Hypopharynx	7

*Tumour stage*	
T1	6
T2	8
T3	2
T4	7
TX	1

*Nodal status*	
N0	11
N1	4
N2	9

*Alcohol history*	
Yes	9
No	10
Prior	3
Unknown	2

*Smoking history*	
Yes	13
No	3
Prior	5
Unknown	3

*Status at day of blood draw*	
No evidence of disease	14
Active disease (presurgery)	10

SCCHN=squamous cell carcinoma of the head and neck.

**Table 2:** Percentages of T_reg_ in PBMC of selected patients with SCCHN by expression of Foxp3 or GITR

**Patient**	**CD4+CD25^high^ (%)**	**CD4+CD25^high^GITR+ (%)**	**CD4+CD25^high^Foxp3+ (%)**
1.	9^a^	4^a^	6^b^
2.	5	5	5
3.	8.5	5	7.5
4.	6.5	3	6
5.	7.0	5	5

Mean±s.d.	7.0±1.5	5.0±1.0	6.0±1.3

a The percentages of T_reg_ were determined upon backgating on CD3+CD4+CD25^high^ cells following staining from surface markers, as described in Materials and Methods.

b The percentages of Foxp3+ cells were determined as indicated above but following cell permeabilisation and intracytoplasma staining.

SCCHN=squamous cell carcinoma of the head and neck; GITR=glucocorticord-induced TNF receptor; PBMC=peripheral mononuclear cells.

**Table 3:** Expression of the TCR-*ζ* chain in T-cell subsets of patients with SCCHN and normal controls^a^

	**CD4+CD25+**	**CD4+CD25^−^**	**CD8+**	**CD4+**
	**TCR-*ζ* chain expression (log MFI)**			
NC	3.7±1.7	4.2±1.8	3.5±1.5	4.3±1.9
SCCHN	3.2±0.7	3.4±0.7	2.8±0.6	3.4±0.7

a Expression of *ζ* was measured in the various T-cell subsets by flow cytometry as described in Materials and Methods. The data are means±s.d.
